# Using ClinicalTrials.gov to Supplement Information in Ophthalmology Conference Abstracts about Trial Outcomes: A Comparison Study

**DOI:** 10.1371/journal.pone.0130619

**Published:** 2015-06-24

**Authors:** Roberta W. Scherer, Lynn Huynh, Ann-Margret Ervin, Kay Dickersin

**Affiliations:** 1 Department of Epidemiology, Johns Hopkins Bloomberg School of Public Health, Baltimore, Maryland, United States of America; 2 Analysis Group, Inc., Boston, Massachusetts, United States of America; Canadian Agency for Drugs and Technologies in Health, CANADA

## Abstract

**Background:**

Including results from unpublished randomized controlled trials (RCTs) in a systematic review may ameliorate the effect of publication bias in systematic review results. Unpublished RCTs are sometimes described in abstracts presented at conferences, included in trials registers, or both. Trial results may not be available in a trials register and abstracts describing RCT results often lack study design information. Complementary information from a trials register record may be sufficient to allow reliable inclusion of an unpublished RCT only available as an abstract in a systematic review.

**Methods:**

We identified 496 abstracts describing RCTs presented at the 2007 to 2009 Association for Research in Vision and Ophthalmology (ARVO) meetings; 154 RCTs were registered in ClinicalTrials.gov. Two persons extracted verbatim primary and non-primary outcomes reported in the abstract and ClinicalTrials.gov record. We compared each abstract outcome with all ClinicalTrials.gov outcomes and coded matches as complete, partial, or no match.

**Results:**

We identified 800 outcomes in 152 abstracts (95 primary [51 abstracts] and 705 [141 abstracts] non-primary outcomes). No outcomes were reported in 2 abstracts. Of 95 primary outcomes, 17 (18%) agreed completely, 53 (56%) partially, and 25 (26%) had no match with a ClinicalTrials.gov primary or non-primary outcome. Among 705 non-primary outcomes, 56 (8%) agreed completely, 205 (29%) agreed partially, and 444 (63%) had no match with a ClinicalTrials.gov primary or non-primary outcome. Among the 258 outcomes partially agreeing, we found additional information on the time when the outcome was measured more often in ClinicalTrials.gov than in the abstract (141/258 (55%) versus 55/258 (21%)). We found no association between the presence of non-matching “new” outcomes and year of registration, time to registry update, industry sponsorship, or multi-center status.

**Conclusion:**

Conference abstracts may be a valuable source of information about results for outcomes of unpublished RCTs that have been registered in ClinicalTrials.gov. Complementary additional descriptive information may be present for outcomes reported in both sources. However, ARVO abstract authors also present outcomes not reported in ClinicalTrials.gov and these may represent analyses not originally planned.

## Introduction

Researchers depend on randomized controlled trial (RCT) findings reported in publications to help inform the design of future studies and assess the effectiveness and harms of a health intervention. Systematic reviewers synthesize the findings from similar but separate trials to estimate an average effect of an intervention; several factors threaten the validity of that estimate, potentially affecting many stakeholders–clinicians, funders, researchers, patients and policy makers [1–3]. For example, there may be failure to report all or some of the findings from an RCT. Similarly, a delay in reporting clinical trials results affects a summary estimate, especially when positive results are published more quickly than null or negative findings.

Chalmers and Glasziou identified four places where waste can occur in the research enterprise, including lack of “accessible full publications” and “unbiased and usable reports” [1]. Systematic reviewers are no longer restricted to information published in peer-reviewed journals, however. They can find information about clinical trials from published protocols, conference abstracts, clinical trial registries, regulatory authority websites, and other sources, although linkages between these sources are often less than optimal [4, 5]. Having to search multiple sources for every trial, and often finding conflicting information among sources when there are multiple reports of the same trial, also constitutes considerable waste.

Trial registration is increasingly being required for many trials for publication, by regulatory bodies [6], or local research ethics boards [7]. In 2004, the International Committee of Medical Journal Editors (ICMJE) announced that publication in ICMJE member journals would be permitted only if the clinical trials had been registered in one of a few selected registers [8]. In 2007 under the United States Food and Drug Administration (FDA) Amendment Acts (FDAAA), sponsors of all trials testing drugs, biologics, and device trials under an investigational new drug application have been required to register within, and update ClinicalTrials.gov for approved drugs and devices within 12 months of study completion to include primary, principal secondary, and safety outcomes from the trial results. However, reporting results from all outcomes named in the protocol is not required [6] nor do all registered trials post results [9, 10]. Currently, about one third of systematic reviewers access clinical trial registries to identify trials for inclusion in systematic reviews [11] even though there is evidence that ClinicalTrials.gov is a rich source of information about study design and execution [12].

Conference abstracts with no associated full publication may provide additional information for a systematic review and are often a source of unpublished, null and negative trial results. Because about 40% of RCT results initially reported in conference abstracts are not published in full, even after 9 years [13], the Institute of Medicine recommends searching for conference abstracts when conducting a systematic review [14]. On the other hand, systematic reviewers have been wary of including information from conference abstracts because of concerns about the paucity and accuracy of information on the population, interventions compared and outcomes, as well as design characteristics that could affect the risk of bias [15].

Another way to look at the problem, however, is that if conference abstracts and trials registers inform us about the existence of unpublished trials we might otherwise not have known about, then trial registry information could supplement the conference abstract information. Conversely, conference abstracts could provide results for outcomes or results that are not reported in a trials register.

For our study, we selected abstracts presented at the Association for Research in Vision and Ophthalmology (ARVO) conference because any conference abstract submitted to ARVO from 2007 onward and describing trial findings is required to be registered. We found that the majority of RCTs described in ARVO abstracts and that reported trial registration were in fact registered with ClinicalTrials.gov [16]. Our objective was to examine in detail the description of the primary and non-primary outcomes reported in the ARVO abstract, and to compare these outcomes with those reported in ClinicalTrials.gov. Our goal was to determine whether outcome information from the trial registry could supplement incomplete data on outcomes described in conference abstracts, and to assess the presence and types of discrepancies in outcomes reported between the two sources.

## Methods

### Identification of randomized controlled clinical trials

We previously described the methods for identification of reports of RCTs presented in conference abstracts at annual ARVO meetings from 2007 through 2009 [16]. Briefly, conference proceedings were hand searched for abstracts of reports of RCTs using the definition for RCT provided in the Handsearching Training Manual of the Cochrane Collaboration [17]. Classification of the study described in each abstract as an RCT was verified by a second person.

The trial registration number included in the abstract was entered in the search function of the ClinicalTrials.gov website (at http://www.clinicaltrials.gov). We classified a trial registration number as valid if there was a corresponding record in ClinicalTrials.gov. We excluded abstracts where the ClinicalTrials.gov record did not describe a population with an eye condition or a population in which one of the trial goals was treatment of or prevention of an eye condition. We also excluded abstracts that described secondary analyses of trial data, nested case-control studies, ancillary studies, and methodological studies associated with an RCT, and abstracts where the ClinicalTrials.gov report clearly stated that the assignment to treatment was not randomized. For RCTs with valid registration numbers, the tabular view of the ClinicalTrials.gov record was accessed on the Internet and hardcopies printed during May and June 2009. If a single trial, as designated by a single ClinicalTrials.gov registration number, was identified in multiple abstracts describing trial findings, we used information from all abstracts to compare with the ClinicalTrials.gov record.

### Data abstraction

Two persons (RWS and LH) independently extracted similar information on study design characteristics from both the abstract and from the ClinicalTrials.gov report on pre-tested paper data collection forms as previously reported [18]. All data were extracted from the ARVO abstract before being extracted from the ClinicalTrials.gov report. Outcome data were collected by recording the outcome(s) exactly as written in both sources and recording whether each outcome was classified as a primary or non-primary outcome. For ARVO abstracts, we defined a primary outcome to be any outcome explicitly described as a primary outcome; all other outcomes named in the abstract were classified as non-primary outcomes. For ClinicalTrials.gov records, we defined only the outcome included in the Primary Outcome field in the tabular view of the record as a primary outcome; all other outcomes named in the ClinicalTrials.gov record were classified as non-primary outcomes, including those reported as secondary or safety outcomes. Discrepancies in data abstraction were resolved by consensus between the two abstractors.

Because there were multiple outcomes reported in both the abstract and ClinicalTrials.gov, we listed each outcome reported in the abstract for a study and then compared each separately with the list of outcomes for that study as reported in ClinicalTrials.gov. We first assessed agreement between each outcome and the primary outcome recorded in ClinicalTrials.gov. If there was no match, we then assessed agreement with each non-primary outcome listed in ClinicalTrials.gov. Agreement was classified as complete agreement, partial agreement, or no agreement. We defined complete agreement as outcomes that were either identical word-for-word, or had synonymous or almost the same wording. For example, we considered the primary outcome in the abstract written as “clinical cure (clinical cure—score 0 for bulbar conjunctivitis, infection and purulent discharge at day 9)” to be synonymous with the primary outcome in ClinicalTrials.gov “clinical cure on test of cure visit at day 9, defined as score of 0 for bulbar conjunctival infection and a score of 0 for conjunctival purulent discharge”. In another example, we classified pairs as having “partial agreement” when the abstract stated the outcomes was “visual acuity at 6 months using ETDRS charts” while the ClinicalTrials.gov record stated simply “visual acuity”; we recorded that additional information was provided by the abstract (i.e., “at 6 months using ETDRS charts”). We classified the additional outcome information by time point when outcome was measured or ‘other’ (for examples see S1 Table. Examples of classification of primary outcomes). If no outcome that was listed in ClinicalTrials.gov matched an outcome reported in the abstract, or if there was no outcome reported in the ClinicalTrials.gov record, we classified the abstract outcome as a “new” outcome. Comparisons were completed in one direction only (i.e., abstract to ClinicalTrials.gov) because it would not be reasonable to expect an abstract to include all outcomes reported in ClinicalTrials.gov. Discrepancies in classification of agreement between the two data abstractors were resolved by consensus.

### Data Analyses

Because we had no *a priori* hypothesis, we did not perform a sample size calculation. All data were entered into an Access 2010 database (Microsoft, Inc.). Analyses, including descriptive analyses and χ^2^ tests to examine differences in proportions, were performed using SAS, version 9.3 (SAS Institute, Carey, NC).

## Results

**Fig 1:**
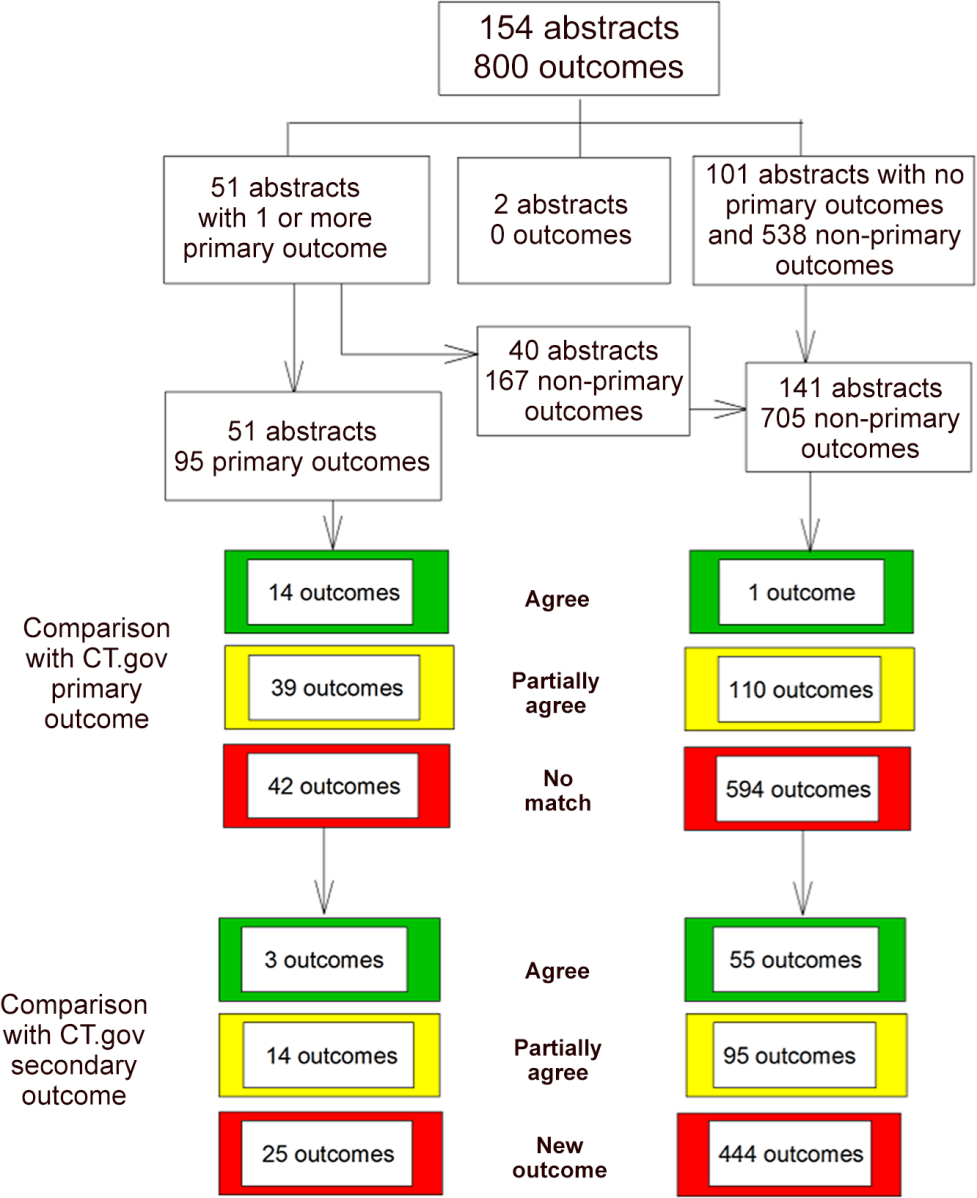
Flow chart. The flow chart shows the number of outcomes by abstract and level of agreement with outcomes in the ClinicalTrials.gov register. Primary outcomes are shown on the left and non-primary outcomes on the right. Both primary and non-primary outcomes were compared with the outcome in the Primary Outcome Field of ClinicalTrials.gov first. Unmatched outcomes were then compared with all other outcomes reported in the ClinicalTrials.gov record. The number of abstract outcomes that completely agreed with the outcome in ClinicalTrials.gov is shown in green, that partially agreed is shown in yellow and that were not matched to any outcome in red. CT.gov = ClinicalTrials.gov

**Fig 2:**
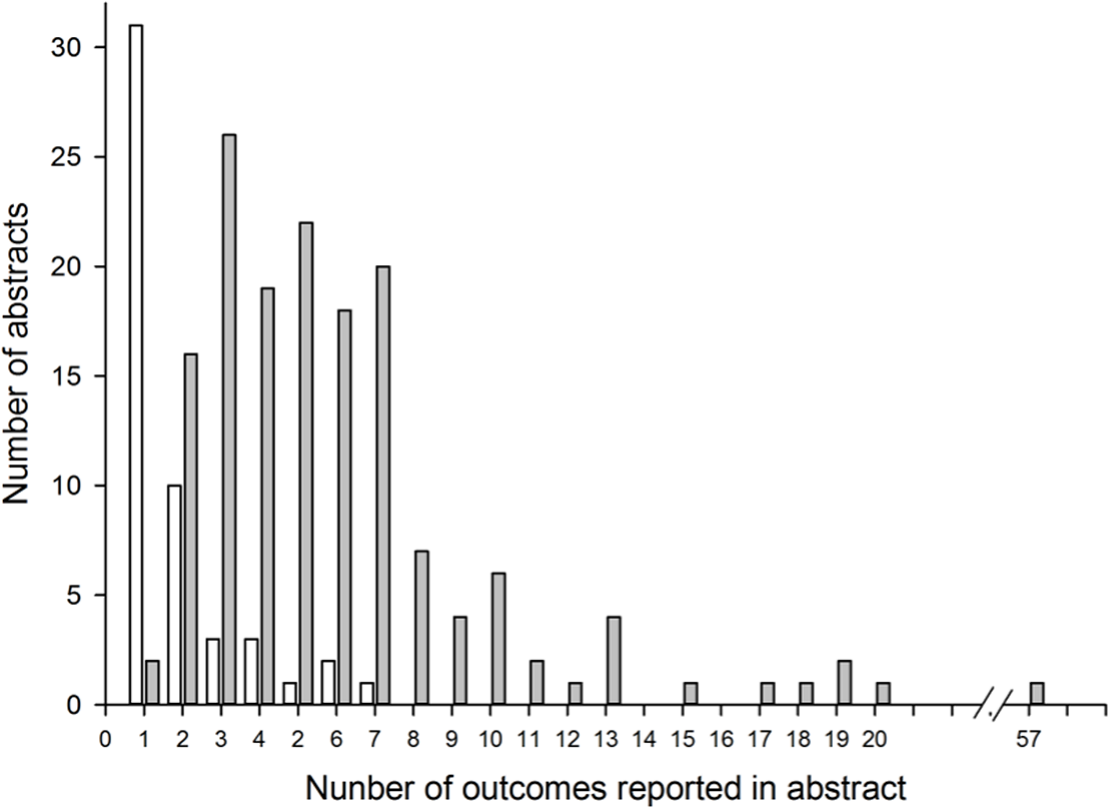
Distribution of primary and non-primary outcomes. The number of primary outcomes (n = 95) that were reported in an abstract are shown in gray bars and non-primary outcomes (n = 705) in white bars.

We identified 496 ARVO conference abstracts describing results of an RCT in an eye population, 154 of which had a matching registration record in ClinicalTrials.gov (see S2 Table. Reasons for exclusion of abstracts from ClinicalTrials.gov–abstract pairs). We identified 800 outcomes in 152 abstracts; there were no outcomes reported in 2 abstracts. We classified 95 outcomes in 51 abstracts as primary outcomes and 705 outcomes in 141 abstracts as non-primary outcomes (see Fig 1). Eleven abstracts reported only one or more primary outcomes, 40 reported both primary and non-primary outcomes, and 101 reported only non-primary outcomes. Typically, there was a single primary outcome reported per abstract (median = 1), although we observed as many as 7 primary outcomes in a single abstract. The distribution of non-primary outcomes reported within a single abstract ranged from 1 to 57 (median = 4) (Fig 2).

### Primary outcomes

**Fig 3:**
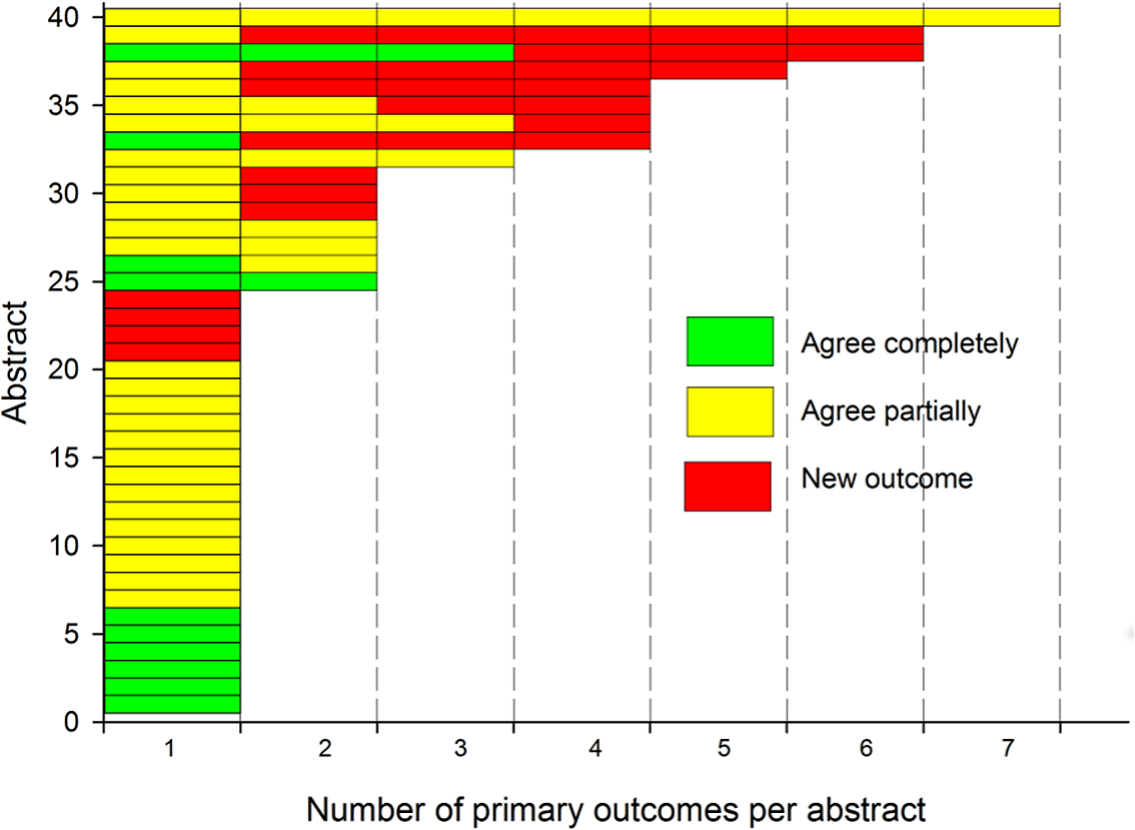
Agreement between primary outcomes reported in abstracts with primary outcome reported in ClinicalTrials.gov. Abstract outcomes from 40 abstracts reporting primary outcomes that completely agree with the outcome in ClinicalTrials.gov are shown in green, those partially agree are shown in yellow, and those that do not match any outcome are in red.

There was an entry in the “Primary Outcome” field of ClinicalTrials.gov for 80.5% (124/154) of ClinicalTrials.gov records, and information on the time point when the primary outcome was to be measured for 72% of them (89/124). There was no primary outcome reported in the ClinicalTrials.gov “Primary Outcome” field for 15 of the primary outcomes reported in 11 abstracts. Comparison of the remaining 80 primary outcomes (reported in 40 abstracts), with the primary outcome recorded in ClinicalTrials.gov, resulted in 14 (18%) agreeing completely, 39 (49%) agreeing partially, and 27 classified as not matching (see Fig 1). There did not appear to be any association between the number of primary outcomes reported in the abstract and the level of agreement (Fig 3).

Of the 42 primary outcomes that had no match with a primary outcome recorded in ClinicalTrials.gov, (15 because there was no outcome in the “Primary Outcome” field of the ClinicalTrials.gov record, and 27 because there was no matching primary outcome in that field), 3 agreed exactly and 14 agreed partially with a non-primary outcome in ClinicalTrials.gov; 25 remained unmatched and were classified as “new” outcomes (see Fig 1).

### Non-primary outcomes

Among the 705 non-primary outcomes reported in 141 abstracts, 1 agreed exactly and 110 (16%) agreed partially with the primary outcome reported in ClinicalTrials.gov for that study. Among the remaining 594 outcomes, 55 (8%) agreed exactly and 95 (13%) agreed partially with a secondary or safety outcome reported in ClinicalTrials.gov; 444 (63%) were not reported in ClinicalTrials.gov and were classified as “new” outcomes (see Fig 1).

Altogether, across the 152 abstracts reporting any outcome, 96 (63%) reported an outcome that agreed exactly (13/152; 9%) or partially (91/152; 60%) with a primary outcome recorded in ClinicalTrials.gov, while all abstracts had at least one outcome that agreed exactly (37/152; 24%) or partially (115/152; 76%), with a primary or secondary outcome recorded in ClinicalTrials.gov. However, 111 abstracts (73%) had at least one outcome that had no matching outcome in ClinicalTrials.gov.

### Factors related to reporting ‘new’ outcomes

We explored factors that could be associated with the presence of a “new” primary outcome or non-primary outcome. We first looked at information on time of initial trial registration. The date of trial registration ranged from 1999 to 2008, with a median registration date of November 2006. Twenty-six of 152 trial records in ClinicalTrials.gov (i.e., the 152 abstracts reporting at least one outcome) had not updated the original trial registration. Examining updated records only, the mean (SD) and median time from registration to the most recent update was 1.58 (1.12) years and 1.58 years, respectively. We found no association between the presence of a “new” outcome and either of these measures of registration time (data not shown).

We also explored whether there was an association between industry sponsorship or multi-center status and the presence of a “new” outcome. Eighty-five trials (56%) received industry sponsorship as noted in either the abstract or the ClinicalTrials.gov record; there was no statistically significant association between industry sponsorship and the presence of a new outcome; 58% (65/111) of abstracts with a new outcome versus 49% (20/41) without a new outcome had industry sponsorship (OR = 1.48; 95% CI 0.72 to 3.04). Multicenter status was likewise not associated with a new outcome (40/111 (36%) versus 15/41 (37%); OR = 0.98; 95% CI = 0.47 to 2.06).

### Outcome descriptors of partially agreeing outcomes

Further examination of the 39 primary outcomes that we classified as partially agreeing showed that although these agreed by domain, additional information related to the time point when the outcome was measured or ‘other’ information (i.e., measurement method, aggregation method, or specific metric) was present in one source but not the other (for examples of the type of “other” information reported in abstract of ClinicalTrials.gov see S1 Table. Examples of classification of primary outcomes). We found additional outcome information in the abstract for 19 (49%), in ClinicalTrials.gov for 17 (44%), and in both sources for 3 (8%) primary outcomes. Of the 205 non-primary outcomes that were in partial agreement, 110 (54%) were described in more detail in ClinicalTrials.gov, and 64 (32%) in more detail in the abstract. The remaining 31 (15%) outcomes in 21 pairs classified as partially agreeing had non-overlapping additional information in both sources. Table 1 displays the type additional information that was available in each source classified by time point or ‘other’ and shows that more information in ClinicalTrials.gov was present on the time point when an outcome was measured. The occurrence of ‘other’ information was about the same for both sources.

**Table 1:** Additional elements^*^ present in partially agreeing outcomes by source.

Additional elements *	Source	
	Abstract	ClinicalTrials.gov
**Time point**	30	93
**Other**	67	31
**Both time frame and other**	25	48
**Total**	**122**	**172**

*Five elements defining an outcome are: domain, (i.e., what is being measured), time point at which the outcome is measured, method of measurement (e.g., Snellen chart), method of aggregation (e.g., change from baseline), and specific metric (e.g., mean) [19, 20]. Additional elements describing the outcome may have been present in the abstract, ClinicalTrials.gov, or both for RCTs with the same domain. They are described here as time point and ‘other’, which includes method of measurement, method of aggregation and specific metric.

## Discussion

In our study we found that among 152 ARVO abstracts reporting an outcome, 63% (96/152) reported at least one outcome that matched a primary outcome in the ClinicalTrials.gov report, and all abstracts reported at least one outcome that matched completely or partially with either a primary or non-primary outcome reported in the ClinicalTrials.gov record. Overall, 56% (53/95) of outcomes described as a primary outcome in the abstract were also described as a primary outcome in ClinicalTrials.gov.

The original goal of this study was to see if the design information recorded in ClinicalTrials.gov could supplement the limited information found in an ARVO conference abstract, facilitating inclusion of conference abstracts in a systematic review when there is no corresponding full publication or an outcome was reported in the abstract that a systematic reviewer wanted to include. The reverse might also be important, i.e. the information in a conference abstract may complement what is recorded in a trials register. ClinicalTrials.gov often has no results [10], and thus conference abstracts may be a unique source of results for unpublished studies. Thus, the conference abstract is a more useful resource that it might have been without a trials register. Conversely, because ClinicalTrials.gov is likely to have more detailed design information than a conference abstract [18], it is a more useful resource for systematic reviewers than it might have been without a conference abstract.

Elements that are used to describe a trial outcome include the domain (i.e., what is being measured), time point at which the outcome is measured, method of measurement (e.g., Snellen chart), method of aggregation (e.g., change from baseline), and specific metric (e.g., mean) [19, 20]. Many outcomes reported in the ARVO abstract only partially agreed with an outcome found in ClinicalTrials.gov, and always by outcome domain. Other elements were inconsistently reported both in the ARVO abstract and in the ClinicalTrials.gov record. For example, among the primary outcomes reported in ClinicalTrials.gov, there was no associated time point when the outcome was measured in 28% (35/124) of the cases with an entry in the “Primary Outcome” field. On the other hand, when the outcome domain reported in the abstract matched that in ClinicalTrials.gov, we found the associated time point in ClinicalTrials.gov more often than in the abstract. This reporting pattern may be due to the requirement by ClinicalTrials.gov to include the time point when reporting outcomes. In contrast, other elements (method of measurement, aggregation, or specific metric) were reported in one or the other sources with about equal frequency. Current recommendations as stated by Zarin [20] are that ClinicalTrials.gov “parallel the level of specificity described in the protocol”, suggesting that all five elements defining an outcome, as described above, should be included in the trials register record.

Based on historical patterns [13], we expect that about 40% of the RCTs described in the ARVO abstract will not be published in full. Although we did not look for associated full publications systematically, we accessed the ClinicalTrials.gov report for each RCT described in this report to look for posted publications and study results. As of 12 January 2015, there was at least one full length publication listed for 42% (64/154), and results posted for 23% (35/154) of RCTs described in the ARVO abstracts included in this report in ClinicalTrials.gov. Thus, trial results may be available for only about half (81/54; 53%) of the RCTs described in this report. RCT results were not required to be posted in ClinicalTrials.gov until 2009, however, so systematic reviewers who want to include an RCT that is neither published nor has results posted can obtain more information by accessing both the ARVO abstract and ClinicalTrial.gov than either one alone. We intend to continue to examine the RCTs reported here by systematically identifying all full length publications specifically reporting the results presented in the abstract and comparing the results described in the abstract with those presented in the full length publication and in ClinicalTrials.gov, if present.

We found that about three quarters of ARVO abstracts reported one or more primary or non-primary outcomes that could not be found in the ClinicalTrials.gov record. The large proportion of “new” outcomes reported in abstracts but not found in ClinicalTrials.gov, raises concerns about the possibility of selective outcome reporting in the abstract. This finding is in agreement with investigators who compared outcomes reported in abstracts, trial protocols, full-text publications and registries [21–31]. For example, Killeen et al found that 12.5% of papers describing a surgical RCT have a new primary outcome and for 21.9% of primary outcomes, the timing was different between the published paper and the registry [29]. Norris et al compared outcomes reported in trials within two comparative effectiveness reviews and found that the most common type of selective outcome reporting was the presence of an outcome not pre-specified in the corresponding trials register (83% and 58% of trials within the two reviews) [32]. Selective outcome reporting in publications remains highly prevalent despite efforts to improve transparency. Mathieu et al. reported that approximately one-third of the studies that he examined had some discrepancies between outcomes reported in the trial registry and outcomes in the publication [28]. Other investigators have reported similar discrepancies between study design and outcomes reported in conference abstracts and full length publications [30, 31]. These studies illustrate the presence of incomplete linkages between sources of information about RCTs, including trial registries, full length publications, abstracts, or other sources, highlighting the level of waste in the research enterprise.

Mathieu et al also showed a higher likelihood of reporting outcomes when results were statistically significant or when industry sponsorship was declared and outcomes favored industry’s drug In addition [28]. We did not find an association between the presence of a new outcome and industry sponsorship. Partly, this may be because our definition of industry sponsorship was quite broad, including a positive report of any type of industry sponsorship in either the ARVO abstract or a report of industry affiliation in the ClinicalTrials.gov record.

Rather than selective outcome reporting, our findings may be due to the trial registration process. Instructions for registration at the ClinicalTrials.gov website defines which outcomes are to be entered, including primary, secondary, and “other pre-specified” outcomes. This latter category is defined in the ClinicalTrials.gov website as “Any other measurements, excluding *post hoc* measures, that will be used to evaluate the intervention(s)”[33]. Any outcome that was not pre-specified might legitimately not be included in the ClinicalTrials.gov register, and contribute to the presence of “new” outcomes. For example, investigators may not be able to register specific harms outcomes simply because the adverse event is unknown at the time of registration, but report these adverse events in conference abstracts. It would be of value for ClinicalTrials.gov to require inclusion of all outcomes in the future, noting which were pre-specified and which were added after trial inception. Alternatively, investigators’ failure to update the required primary and secondary outcomes entered in the trial registry may contribute to the presence of “new” outcomes. Since it is allowed, investigators may not be entering all pre-specified RCT outcomes in the ClinicalTrials.gov record, perhaps with outcomes reported in the conference abstracts deemed to be less important or ancillary. Because non-primary outcomes are not used to determine sample size and contribute to power calculations for trials, they may be considered less important by study investigators. On the other hand, a non-primary outcome may be considered a reasonable topic for presentation at a conference. Another possible reason for our finding of “new” outcomes in the ARVO abstract is that investigators may have elected to submit an abstract describing a *post hoc* outcome solely for the purpose of attendance at a conference. Currently, a ClinicalTrials.gov report serves as a proxy for a study protocol. However, comparison of the data elements recommended by SPIRIT for reporting trial protocols is much more detailed that a ClinicalTrials.gov report [34] and this includes reporting of all outcomes.

Our findings beg the question of what systematic reviewers should do when outcome information is recorded on both an abstract and ClinicalTrials.gov and the information does not agree. Certainly, a systematic reviewer would want to report the result of any outcome from an abstract with a matching outcome (primary or non-primary) in ClinicalTrials.gov. However, when the outcomes differ, systematic reviewers should consider how to proceed. For “new” outcomes not reported in ClinicalTrials.gov, a first step would be to investigate whether there is a corresponding full length publication or publically available protocol. Alternatively, the systematic reviewer could request information directly from the study investigators. In cases where these options are not available, and the outcome of interest is not listed in ClinicalTrials.gov, the systematic reviewer has the option of following the IOM recommendation to include trial results reported only in a conference abstract. Because results from abstracts that remain unpublished are less likely to show positive effects of a study intervention[13], it would be prudent for the systematic reviewer to recognize this potential limitation and conduct a sensitivity analysis by comparing meta-analyses with and without abstract results. Regardless, the implications of including outcome findings reported only in abstracts should be discussed.

Several limitations of our study should be noted. Because we only looked at abstracts from ARVO, our study is limited to vision research and applicability of our findings to other areas of healthcare is uncertain. Other areas of healthcare may not have the proportion of “new” primary or non-primary outcomes reported in abstracts that we observed for ARVO abstracts. Secondly, we looked at abstracts that were presented in 2007, 2008 and 2009, and the trials they describe were registered at ClinicalTrials.gov prior to those dates. Instructions for registration when these trials were registered were not as explicit as 2015 instructions nor were quality control measures as rigorous. In 2013 there was still concern about the level of detail required for describing an outcome in ClinicalTrials.gov, however [20]. Despite these limitations, the study suggests that supplementing information from ClinicalTrials.gov with results data presented in conference abstracts offers a viable alternative for obtaining more complete information about unpublished trials for use in systematic reviews than from either source alone.

## Supporting Information

S1 Table Examples of classification of primary outcomes. (DOC)

S2 Table Reasons for exclusion of abstracts from ClinicalTrials.gov–abstract pairs. (DOC)
