# Supplementary material for: Using ClinicalTrials.gov to Supplement Information in Ophthalmology Conference Abstracts about Trial Outcomes: A Comparison Study
Source: PLoS One. 2015 Jun 24;10(6):e0130619. doi: 10.1371/journal.pone.0130619 (PMC4479484; doi:10.1371/journal.pone.0130619)
Supplement: S1 Table — (DOC) [file pone.0130619.s001.doc]

**Supplementary Information**

**Table 1. Examples of classification of primary outcomes.** Matching information present in the abstract and the ClinicalTrials.gov report is bolded.

|  | **Abstract outcome** | **CT.gov primary outcome** | **Classification** |
| --- | --- | --- | --- |
|
| **1** | **Visual acuity** (ETDRS letters) | **Visual acuity** at 18 months | Partial agreement |
|  | Number of treatments |  | New outcome |
|  |  |  |  |
| **2** | **Visual acuity** | **Visual acuity** | Complete agreement |
|  | **Mean change in lesion size** | **Mean change in lesion size** | Complete agreement |
|  | **Mean change in foveal thickness** | **Mean change in foveal thickness** | Complete agreement |
|  | Retreatment rate |  | New outcome |
|  | Incidence of adverse events |  | New outcome |
|  | Severity of adverse events |  | New outcome |
|  |  |  |  |
| **3** | **Visual acuity** (ETDRS letters) | Mean change from baseline in best corrected **visual acuity** score at 6 months | Partial agreement |
|  |  |  |  |
| **4** | **Proportion of subjects who gain ≥15 letters in best corrected visual acuity compared to baseline at 24 months** | **Proportion of subjects who gain at least 15 letters in best corrected visual acuity compared with baseline at 24 months** | Complete agreement |
|  |  |  |  |
| **5** | Changes in central **macular thickness over a 12-month** follow-up | **Macular thickness** measured by OCT **at** **12 months** | Partial agreement |
|  | Changes in visual acuity over a 12-month follow-up |  | New outcome |
|  |  |  |  |
| **6** | **Pain levels** reported at 2 hours post-op days 1 through 4 | **Level of pain** assessed using the visual analog scale of pain | Partial agreement |
|  |  |  |  |
| **7** | **Retinal thickness** in the peak oedematous field **on the retinal maps of the fast module scans of the Stratus OCT** | **Retinal thickness by fast retinal thickness mapping by optical coherence tomography** at 0,2,4,8,10,15,20,30,60,90,120, and 180 min after last swallow of glycerol | Partial agreement |
|  |  |  |  |
| **8** | ETDRS **best corrected visual acuity** at 2 weeks | **Best corrected visual acuity** | Partial agreement |
|  | ETDRS **best corrected visual acuity** at 4 weeks | **Best corrected visual acuity** | Partial agreement |
|  | ETDRS **best corrected visual acuity** at 12 weeks | **Best corrected visual acuity** | Partial agreement |
|  | **OCT** at 2 weeks | **OCT** foveal thickness | Partial agreement |
|  | **OCT** at 6 weeks | **OCT** foveal thickness | Partial agreement |
|  | **Fluorescein angiography** at 2 weeks | **Fluorescein angiogram** | Partial agreement |
|  | **Fluorescein angiography** at 6 weeks | **Fluorescein angiogram** | Partial agreement |
