# Supplementary material for: Using ClinicalTrials.gov to Supplement Information in Ophthalmology Conference Abstracts about Trial Outcomes: A Comparison Study
Source: PLoS One. 2015 Jun 24;10(6):e0130619. doi: 10.1371/journal.pone.0130619 (PMC4479484; doi:10.1371/journal.pone.0130619)
Supplement: S2 Table — (DOC) [file pone.0130619.s002.doc]

**Supplementary Information**

**Table 2.** **Reasons for exclusion of abstracts from ClinicalTrials.gov–abstract pairs.** Of 496 abstracts that were identified as describing a randomized clinical trial, 154 were matched with a ClinicalTrials.gov record and included in this study and 341 were excluded.

| **Reasons for exclusion** | **Number excluded** |
| --- | --- |
| No registration number | 160 |
| Not registered at ClinicalTrials.gov | 60 |
| Pending or bad registration | 15 |
| Multiple registration numbers or multiple RCTs described in single abstract | 16 |
| Description of secondary or methodologic data only | 60 |
| Duplicate registration numbers | 27 |
| Classified as “not randomized” in ClinicalTrials.gov | 4 |
